# Paravertebral muscle degeneration-guided selective fusion strategy for degenerative lumbar scoliosis: a novel approach to minimize surgical invasiveness while optimizing clinical outcomes

**DOI:** 10.3389/fmed.2026.1760118

**Published:** 2026-06-02

**Authors:** Qi Guo, Jianliang Chen, Huahai Yao, Zhe Wu, Jixin Chen, Yong Xu, Jiafeng Zhang, Yingzhou Li

**Affiliations:** 1 Zhejiang Chinese Medical University, Hangzhou, China; 2 Department of Orthopedics, Shaoxing Shangyu Traditional Chinese Medicine Hospital, Shaoxing, China

**Keywords:** degenerative lumbar scoliosis, Goutallier classification, minimally invasive strategy, multifidus, paravertebral muscles, proximal junctional kyphosis, selective fusion

## Abstract

**Background:**

Proximal junctional kyphosis (PJK) and proximal junctional failure (PJF) remain unresolved complications in degenerative lumbar scoliosis (DLS) surgery. This study evaluated a PVM degeneration-guided selective fusion strategy in which fusion levels were determined according to pre-operative paravertebral muscle (PVM) degeneration patterns.

**Methods:**

This retrospective cohort study included 22 patients with DLS treated between January 2022 and December 2024 (mean follow-up 28.4 ± 3.8 months, range 24–36 months). Fusion-degeneration concordance was defined as exact alignment between fusion boundaries and the contiguous block of significantly degenerated PVM segments (Goutallier grade ≥3). PJK was defined as a proximal junctional angle ≥10° and at least 10° greater than the pre-operative measurement. PJF was defined as PJK associated with vertebral fracture, instrumentation failure, subluxation, or requiring revision surgery. The radiographic correction maintenance rate was calculated as [(immediate post-operative correction amount – final follow-up correction loss) / immediate post-operative correction amount] × 100%. The primary outcome was the incidence of PJK/PJF at final follow-up. Secondary outcomes included radiographic correction maintenance and patient-reported outcomes. Longitudinal radiographic changes were analyzed using repeated-measures ANOVA. Multivariable linear regression was performed to identify factors associated with ODI improvement rate.

**Results:**

No cases of PJK or PJF were observed. Mean fusion length was 4.3 ± 1.1 segments, with fusion-degeneration concordance achieved in 19/22 patients (86.4%). Coronal and sagittal alignment improved significantly post-operatively and were maintained at final follow-up (all *P* < 0.001). Correction loss at final follow-up was 1.0 ± 1.2°, corresponding to a maintenance rate of 88.2 ± 7.3%. In multivariable analysis, a greater number of degenerated PVM segments was negatively associated with ODI improvement rate (*B* = −0.11, 95% CI −0.18 to −0.04, *P* = 0.003), whereas fusion-degeneration concordance was positively associated with ODI improvement (*B* = 0.38, 95% CI 0.12 to 0.63, *P* = 0.006).

**Conclusions:**

Selective fusion guided by PVM degeneration patterns represents a paradigm shift from geometry-based to biology-guided surgical planning for DLS. This strategy achieves excellent clinical and radiographic outcomes with reduced fusion extent, surgical trauma, and junctional complications. The approach warrants validation through prospective multicenter trials.

## Introduction

Degenerative lumbar scoliosis (DLS) represents a complex three-dimensional spinal deformity arising *de novo* in adulthood, characterized by lateral curvature exceeding 10°, vertebral rotation, sagittal malalignment, and frequently coexisting central or foraminal stenosis (1, 2). As global demographics shift toward aging populations, DLS prevalence continues to rise, constituting a leading cause of chronic low back pain, ambulatory dysfunction, and diminished quality of life among elderly individuals (3). The surgical management of symptomatic DLS aims to achieve neural decompression, restore spinopelvic alignment, and establish durable stability through arthrodesis.

Conventional wisdom in DLS surgery has favored extensive instrumented fusion, typically spanning 6–8 or more segments, to ensure robust correction and minimize pseudarthrosis risk (4). While this approach reliably achieves radiographic correction, it imposes considerable physiologic burden, including prolonged operative duration, substantial blood loss, extended hospitalization, and elevated healthcare costs (5).More critically, long-segment constructs generate marked mechanical stress concentration at the proximal junction, precipitating proximal junctional kyphosis (PJK) or failure (PJF) in 20–40% of cases—complications associated with significant morbidity and frequent revision surgery (6, 7).

Recent advances in spinopelvic alignment theory and recognition of muscle function in spinal stability have prompted reconsideration of traditional fusion strategies (8, 9). Paravertebral muscles (PVMs), particularly the deep multifidus, function as primary dynamic stabilizers providing segmental control and fine motor coordination (10). Fatty infiltration and atrophy of these muscles—readily quantifiable on T2-weighted MRI using validated grading systems—strongly correlate with lumbar degeneration, instability, and deformity progression (11, 12). Researchers demonstrated that patients developing PJK exhibit significantly poorer proximal PVM quality compared to those maintaining junctional integrity, suggesting a protective role of preserved muscle function (13). Because PJK/PJF remains one of the most consequential and unresolved complications in adult spinal deformity surgery, we prioritized junctional outcomes as the central clinical endpoint of this investigation. In a single-cohort study, the clinical value of PVM degeneration as a planning indicator should therefore be interpreted primarily through its association with junctional integrity and durability of alignment maintenance. We hypothesized that segments with severe PVM degeneration have lost effective dynamic stabilization capacity and represent primary “culprit levels” driving symptoms and deformity progression, whereas segments with preserved PVM quality retain intrinsic stability and may be safely excluded from fusion. This biology-guided paradigm contrasts with purely geometric approaches (curve magnitude, sagittal vertical axis) or mechanical criteria (motion segment translation/angulation) that dominate current surgical planning.

The present study applies a PVM degeneration-guided selective fusion strategy in a consecutive cohort of patients with degenerative lumbar scoliosis (DLS) using a single-institution retrospective design. Previous studies have reported that paraspinal muscle quality—commonly characterized by muscle atrophy and fatty infiltration on MRI-is associated with clinical outcomes and the durability of post-operative alignment in adult spinal deformity, and has been implicated as a potential risk factor for junctional complications including PJK/PJF. These findings support the clinical relevance of incorporating PVM degeneration into surgical decision-making as a biology-guided marker. Therefore, in the absence of a comparator cohort, we positioned the usefulness of PVM degeneration by referencing this established evidence and examined whether a PVM degeneration-guided selective fusion strategy could achieve satisfactory symptom improvement and radiographic correction with acceptable alignment maintenance and junctional safety over follow-up. Given the absence of a comparison group, our evaluation is observational and is intended to examine whether a fusion construct planned primarily according to PVM degeneration patterns can achieve satisfactory symptom relief, radiographic correction, and alignment maintenance while minimizing proximal junctional complications over a minimum 24-month follow-up. Accordingly, the primary endpoint of this study was the incidence of PJK/PJF at final follow-up. Secondary endpoints included patient-reported outcomes (VAS, ODI, SF-36), radiographic parameters (coronal Cobb angle, lumbar lordosis, and sagittal vertical axis), fusion status, and complications. Because alternative fusion-selection strategies were not directly compared, any contextual comparison with prior studies should be interpreted cautiously due to differences in patient selection and surgical indications.

## Materials and methods

### Study design and patient selection

**Figure 1:**
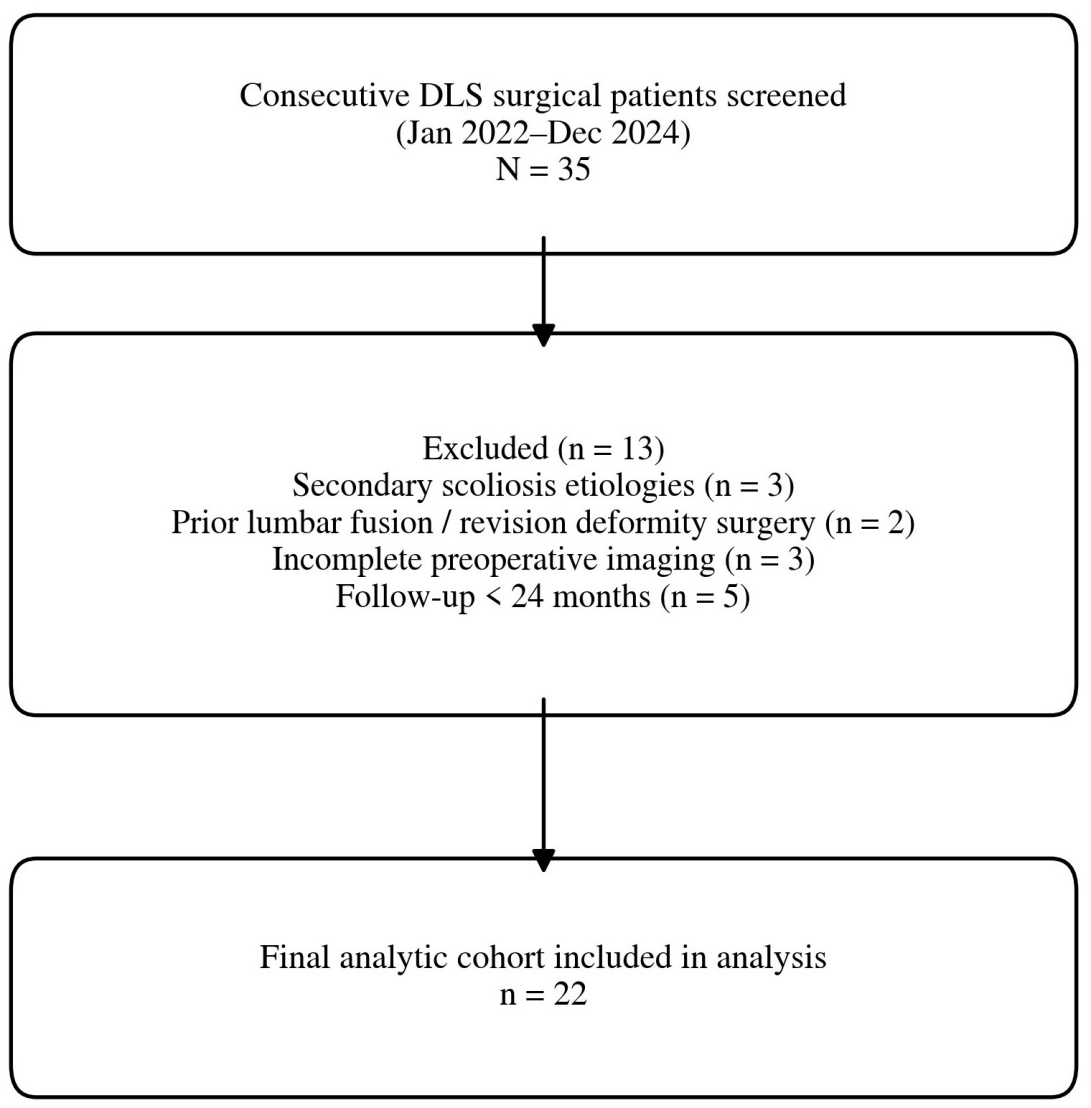
Study flowchart.

This single-institution retrospective cohort study received Institutional Review Board approval. We reviewed all consecutive DLS patients who underwent surgical treatment between January 2022 and December 2024 at our tertiary spinal surgery center. The inclusion criteria were age ≥50 years, a diagnosis of degenerative lumbar scoliosis (DLS) clinically and radiographically with a major curve Cobb angle >10°, persistent disabling axial back pain and/or radicular symptoms despite ≥3 months of comprehensive conservative management (physical therapy, medications, and epidural injections), complete preoperative imaging including standing full-length spine radiographs, lumbar CT, and lumbar MRI, and a minimum 24-month follow-up with complete clinical and radiographic data. Exclusion criteria included secondary scoliosis from identifiable causes (such as trauma, tumor, infection, or tuberculosis), congenital spine deformity, neuromuscular scoliosis, or ankylosing spondylitis, previous lumbar fusion surgery, hip or knee arthroplasty altering spinopelvic alignment, leg length discrepancy >2 cm, severe cardiopulmonary or other comorbidities precluding major surgery, and incomplete follow-up or imaging data. During the study period (January 2022 to December 2024), 35 consecutive patients underwent surgical treatment for DLS at our institution. Of these, 13 were excluded due to secondary scoliosis etiologies (*n* = 3), prior lumbar fusion/revision deformity surgery (*n* = 2), incomplete pre-operative imaging precluding PVM or radiographic assessment (*n* = 3), and insufficient follow-up (< 24 months; *n* = 5). After exclusions, the final analytic cohort consisted of 22 patients. A flow diagram summarizing cohort derivation is provided in Figure 1.

Twenty-two patients (14 males, eight females) met all criteria, with a mean age of 65.4 ± 7.2 years (range: 56–80). Bone mineral density (BMD) was not routinely assessed using a standardized protocol in this retrospective cohort, and dual-energy X-ray absorptiometry (DXA) data were not consistently available for all patients. Therefore, BMD was not included as a baseline variable in the present analyses.

### Pre-operative assessment protocol

#### Clinical evaluation

A standardized clinical examination documented pain characteristics (axial vs. radicular), neurologic deficits (motor weakness, sensory changes, reflex abnormalities), and functional limitations (walking distance, activity restrictions). Symptom severity was quantified using validated instruments described below.

#### Radiographic assessment

##### Plain radiography

Standing posteroanterior and lateral full-spine radiographs (36-inch cassette) captured global alignment. Coronal Cobb angle, lumbar lordosis (LL), sagittal vertical axis (SVA), and pelvic parameters were measured using validated techniques. Coronal Cobb angle was measured on standing posteroanterior radiographs between the superior endplate of the most tilted upper-end vertebra and the inferior endplate of the most tilted lower-end vertebra of the major lumbar curve. LL was defined as the angle between the superior endplate of L1 and the superior endplate of S1 on standing lateral radiographs. SVA was measured as the horizontal offset between the C7 plumb line and the posterosuperior corner of S1. All radiographic measurements were performed using a digital measurement system by two independent assessors who were blinded to clinical outcomes. Lateral bending radiographs assessed curve flexibility. Segments demonstrating “level disc spaces” on coronal views—minimal height variation in flexion-extension-were noted as potentially autofused or intrinsically stable.

##### Computed tomography

Thin-slice (1 mm) multiplanar reconstructions characterized bony anatomy, facet joint arthropathy, foraminal stenosis severity, and vertebral rotation/subluxation at each level.

##### Magnetic resonance imaging

High-resolution 1.5T or 3.0T MRI with T1- and T2-weighted sequences in axial, sagittal, and coronal planes evaluated neural compression and PVM quality.

#### Paravertebral muscle degeneration assessment

PVM fatty infiltration was graded independently by two fellowship-trained musculoskeletal radiologists, each with over 10 years of experience, using the Goutallier classification on axial T2-weighted images at each lumbar level. The grading scale was as follows: Grade 0 represented normal muscle with no fat, Grade 1 indicated some fatty streaks, Grade 2 signified fat less than muscle, Grade 3 represented equal amounts of fat and muscle, and Grade 4 indicated more fat than muscle. Both the multifidus and erector spinae muscles were evaluated bilaterally, with the worse side determining the grade for each segment. In cases of inter-rater disagreements, a third senior radiologist was consulted for adjudication. Inter-rater reliability was assessed using Cohen's kappa coefficient.

#### Reliability assessment

Interobserver and intraobserver reliability were evaluated for radiographic measurements (coronal Cobb angle, lumbar lordosis, and sagittal vertical axis), PVM degeneration grading (Goutallier classification), and fusion grading (Bridwell classification). Two independent observers performed all measurements/grading while blinded to clinical outcomes. For intraobserver reliability, each observer repeated the measurements/grading after a 2-week interval on a randomly selected subset of images/cases, blinded to their initial results. Intraclass correlation coefficients (ICC; two-way random-effects model, absolute agreement) with 95% confidence intervals (CIs) were used for continuous radiographic variables, and weighted Cohen's kappa with 95% CIs was used for ordinal grading systems (Goutallier and Bridwell).

#### Operational definition

Segments with Goutallier grade ≥3 in either muscle group were designated as “significantly degenerated,” indicating >50% fatty infiltration and presumed loss of effective dynamic stabilization capacity based on biomechanical literature [17].

#### Fusion level selection algorithm

Our novel fusion level selection strategy integrated three complementary data sources to guide the decision-making process. First, Mandatory Fusion Levels (Must Include) were identified as segments with significant PVM degeneration (Goutallier ≥3) forming contiguous blocks, segments with symptomatic neural compression requiring decompression, and segments exhibiting dynamic instability on flexion-extension films (translation >3 mm or angulation >10°). Second, Preservation Targets (Preferentially Spare) included segments with preserved PVM quality (Goutallier ≤ 2) and stable kinematics, as well as levels demonstrating minimal motion on dynamic imaging, suggesting potential spontaneous stabilization. Finally, Boundary Refinement Considerations took into account coronal “level disc spaces,” which served as potential endpoints for the construct if they were associated with preserved PVM quality. Additionally, proximal and distal junctional zones required adequate PVM quality (ideally Goutallier ≤ 2) to serve as soft boundaries for the fusion. This comprehensive approach ensured optimal fusion segment selection while minimizing unnecessary disruption to healthy segments.

##### Validation

In all cases, the planned fusion extent covered all contiguously degenerated PVM segments. Extensions beyond this zone were dictated by symptomatic stenosis or mechanical instability not accounted for by muscle status alone. Fusion-degeneration concordance was defined as “yes” when the proximal and distal fusion boundaries exactly matched the contiguous block(s) of significantly degenerated PVM segments (Goutallier ≥3) without inclusion of additional non-degenerated levels. Cases requiring extension beyond the degenerated zone due to symptomatic stenosis or mechanical instability were classified as “no” (non-concordant) for the purpose of regression analyses.

#### Surgical technique

All procedures were performed by a consistent senior surgical team under general anesthesia with multimodal neuromonitoring. A midline posterior approach with subperiosteal dissection to the planned levels was used, employing minimally disruptive techniques to preserve paraspinal attachments at non-fusion segments. Targeted foraminal and lateral recess decompression was carried out to address symptomatic neural compression, while facet joint integrity was preserved except at the intended fusion sites, with undercutting laminectomy techniques to avoid destabilizing total facetectomy. Osteotomy grade was determined by curve flexibility, with Smith-Petersen osteotomies (SPO) used for moderate stiffness, pedicle subtraction osteotomies (PSO) reserved for severe sagittal malalignment with rigid deformity, and Ponte osteotomies facilitating segmental lordosis restoration. Pedicle screws were placed at all levels within the planned construct, with fluoroscopic or navigational confirmation, and transforaminal lumbar interbody fusion (TLIF) was performed at segments with severe PVM degeneration to restore anterior column support and enhance fusion biology. Morselized local autograft was supplemented with allograft in interbody cages and posterolateral gutters, and pre-contoured rods were sequentially compressed and distracted to restore alignment. Bilateral S1 pedicle screws were used when fusion extended to L5-S1, with S2-alar-iliac (S2AI) screws added for enhanced fixation in cases of severe pelvic obliquity or poor bone quality. Small multifidus biopsies (< 1 cm^3^) were harvested from severely degenerated segments at the index level for histopathologic confirmation of imaging findings using hematoxylin-eosin staining.

### Outcome measures

#### Primary clinical outcomes

Standardized questionnaires were administered pre-operatively, on post-operative day 1 (for pain assessment), and at all follow-up visits. Post-operative clinical assessments were performed at post-operative day 1, 3 months, 6 months, 12 months, and at the final follow-up (minimum 24 months). Radiographic follow-up was obtained at post-operative day 1 and at each scheduled follow-up visit, with final follow-up radiographs used for assessment of correction maintenance and junctional complications. The primary outcome was the incidence of PJK and PJF at final follow-up. Secondary outcomes included VAS, ODI, SF-36, radiographic correction/maintenance parameters, fusion status (Bridwell grading), and complications.

#### Secondary radiographic outcomes

Standing radiographs taken on post-operative day 1 and at all follow-up intervals were used to assess the coronal Cobb angle (major lumbar curve), lumbar lordosis (L1-S1), sagittal vertical axis, and construct alignment maintenance. Fusion status was evaluated at the final follow-up using dynamic (flexion-extension) radiographs and CT when indicated. Fusion was graded according to the Bridwell classification: Grade I indicated fusion with remodeling and trabeculae present; Grade II indicated the graft was intact but not fully remodeled, with no lucencies; Grade III indicated the graft was intact with potential lucency at the junction; and Grade IV indicated fusion was absent, with motion or lucency present. Grades I and II were considered successful fusion.

#### Complication assessment

All perioperative and follow-up complications were prospectively recorded and categorized as follows: wound complications, which included superficial and deep infections as well as dehiscence; neurologic complications, such as root injury, dural tear, and CSF leak; medical complications, which encompassed DVT/PE, pneumonia, and UTI; implant-related events, including screw malposition, rod fracture, and pseudarthrosis; junctional complications, with proximal junctional kyphosis (PJK) defined as a proximal junctional angle greater than 10° and at least 10° greater than pre-operative measurements, and proximal junctional failure (PJF) defined as PJK associated with fracture or instrumentation failure; and adjacent segment degeneration.

#### Statistical analysis

Data were analyzed using SPSS version 26.0 (IBM Corp., Armonk, NY). Continuous variables were expressed as mean ± standard deviation (SD), and categorical variables as frequencies and percentages. Normal distribution was verified using the Shapiro-Wilk test. Primary analysis was conducted using repeated-measures ANOVA to compare VAS, ODI, SF-36, and radiographic parameters (coronal Cobb angle, lumbar lordosis, and sagittal vertical axis) across three time points (pre-operative, post-operative day 1, and final follow-up), with *post-hoc* Bonferroni correction for multiple comparisons. nterobserver and intraobserver reliability were assessed for radiographic measurements and grading systems as described above, using ICC for continuous variables and weighted kappa for ordinal variables, both with 95% confidence intervals. A multivariate analysis employing multiple linear regression identified independent predictors of ODI improvement rate, defined as \[(pre-operative ODI – final ODI) / pre-operative ODI] × 100%. Independent variables included age, sex, BMI, pre-operative Cobb angle, number of degenerated PVM segments, fusion segment count, fusion-degeneration concordance (whether fusion exactly matched degenerated segments: yes/no), inclusion of S1 (yes/no), operative time, and blood loss. Multicollinearity was assessed using variance inflation factors (VIF < 5), and the model's goodness-of-fit was evaluated by adjusted *R*^2^. Correlation analyses were conducted as exploratory analyses to examine whether the extent of PVM degeneration was associated with (i) fusion extent (number of fused segments), (ii) baseline symptom severity (VAS, ODI), and (iii) radiographic correction magnitude (e.g., changes from pre-operative to post-operative day 1 and/or to final follow-up). These analyses were intended to support the biological plausibility of using PVM degeneration patterns as an indicator for fusion-level selection rather than to establish causality. Statistical significance was set at *P* < 0.05 (two-tailed).

## Results

### Patient demographics and baseline characteristics

Twenty-two patients completed the study with mean follow-up 28.43 ± .8 months (range: 24–36). Baseline demographics are summarized in Table 1. At final follow-up, no cases of proximal junctional kyphosis or proximal junctional failure were observed. Radiographic measurements demonstrated good-to-excellent reproducibility. For continuous parameters, interobserver reliability was excellent for coronal Cobb angle (ICC = 0.92, 95% CI 0.88–0.95), lumbar lordosis (ICC = 0.89, 95% CI 0.84–0.93), and sagittal vertical axis (ICC = 0.91, 95% CI 0.87–0.94). Intraobserver reliability was similarly excellent for coronal Cobb angle (ICC = 0.94, 95% CI 0.91–0.96), lumbar lordosis (ICC = 0.91, 95% CI 0.87–0.94), and sagittal vertical axis (ICC = 0.93, 95% CI 0.90–0.95). For ordinal grading systems, interobserver agreement was substantial for Goutallier grading (weighted κ = 0.78, 95% CI 0.71–0.85) and Bridwell fusion grading (weighted κ = 0.81, 95% CI 0.74–0.87). Intraobserver agreement was excellent for both Goutallier grading (weighted κ = 0.85, 95% CI 0.79–0.90) and Bridwell fusion grading (weighted κ = 0.87, 95% CI 0.82–0.92). All measurements were performed by two independent, fellowship-trained spine surgeons blinded to clinical outcomes, with repeated measurements conducted after a 2-week interval.

**Table 1:** Patient demographics and baseline characteristics.

Parameter	Value
Demographics	
Age (years), mean ± SD	65.47 ± .2
Female sex, *n* (%)	8 (36.4)
Body mass index (kg/m^2^), mean ± SD	26.33 ± .8
Comorbidities, *n* (%)	
Hypertension	12 (54.5)
Diabetes mellitus	6 (27.3)
**Presenting symptoms**, ***n*** **(%)**	
Axial back pain	22 (100)
Unilateral radiculopathy	11 (50.0)
Bilateral radiculopathy	7 (31.8)
Neurogenic claudication	14 (63.6)
Motor weakness	4 (18.2)
**Baseline clinical scores, mean** ±**SD**	
VAS back pain	5.71 ± .0
VAS leg pain	4.81 ± .3
ODI (%)	79.99 ± .3
SF-36	68.65 ± .3
**Radiographic parameters, mean** ±**SD**	
Cobb angle (°)	27.81 ± 4.1
Lumbar lordosis (°)	24.61 ± 2.3
Sagittal vertical axis (mm)	68.42 ± 8.7
Pelvic incidence (°)	52.89 ± .6

### Paravertebral muscle degeneration assessment

Inter-rater reliability for PVM Goutallier grading was excellent (κ = 0.82, 95% CI: 0.76–0.88, *P* < 0.001), validating the reproducibility of this assessment method. Histopathologic examination of intraoperative multifidus biopsies from 22 index segments confirmed correspondence with pre-operative MRI grading in all cases.

The distribution of significantly degenerated PVM segments (Goutallier ≥3) varied among patients: five patients had three consecutive degenerated segments, nine had four segments, five had five segments, and three had extensive degeneration spanning seven segments (Table 2). Sex-stratified analyses showed no significant differences between males and females in the number of degenerated PVM segments or the number of fused segments.

**Table 2:** Correspondence between PVM degeneration and fusion extent.

Degenerated segments	Patients (*n*)	Representative levels	Concordance
Three consecutive	5	L3–L5, L2–L4	5/5 (100%)
Four consecutive	9	L2–S1, L3–S1	7/9 (77.8%)
Five consecutive	5	L1–S1	4/5 (80.0%)
Seven consecutive	3	T11–S1	3/3 (100%)
Overall	22	—	19/22 (86.4%)

Exploratory correlation analyses are summarized in Table 3. The number of degenerated PVM segments showed a strong positive correlation with the number of fused segments (*r* = 0.94, *P* < 0.001). The number of degenerated PVM segments was also positively correlated with baseline symptom severity, including pre-operative ODI (*r* = 0.658, *P* = 0.001) and pre-operative VAS for back pain (*r* = 0.512, *P* = 0.015). Regarding radiographic correction magnitude, the number of degenerated PVM segments correlated with ΔCobb (Pre-op → POD1; *r* = 0.423, *P* = 0.032), ΔLL (*r* = 0.387, *P* = 0.048), and ΔSVA (*r* = 0.456, *P* = 0.021). In addition, the number of fused segments correlated with ΔCobb (*r* = 0.398, *P* = 0.041) and ΔSVA (*r* = 0.412, *P* = 0.036), whereas its correlation with ΔLL did not reach statistical significance (*r* = 0.361, *P* = 0.062). Three patients required fusion extension beyond the degenerated PVM zone due to symptomatic stenosis or instability and were therefore classified as non-concordant according to the pre-defined criteria.

**Table 3:** Exploratory correlations among PVM degeneration extent, fusion extent, symptom severity, and correction magnitude.

Variable 1	Variable 2	Correlation (*r*/ρ)	*P*-value
No. of degenerated PVM segments	No. of fused segments	0.94	< 0.001
No. of degenerated PVM segments	Pre-operative ODI	0.658	0.001
No. of degenerated PVM segments	Pre-operative VAS (back pain)	0.512	0.015
No. of degenerated PVM segments	ΔCobb (Pre-op → POD1)	0.423	0.032
No. of degenerated PVM segments	ΔLL (Pre-op → POD1)	0.387	0.048
No. of degenerated PVM segments	ΔSVA (Pre-op → POD1)	0.456	0.021
No. of fused segments	ΔCobb (Pre-op → POD1)	0.398	0.041
No. of fused segments	ΔLL (Pre-op → POD1)	0.361	0.062
No. of fused segments	ΔSVA (Pre-op → POD1)	0.412	0.036

PVM, paravertebral muscle; ODI, Oswestry Disability Index; VAS, visual analog scale; POD1, post-operative day 1; Δindicates change (correction magnitude).

### Surgical parameters

Mean operative time was 2,553 ± 7 min (range: 195–340). Mean intraoperative blood loss was 3001 ± 62 ml (range: 150–650). All patients received TLIF at a mean of 2.10 ± .8 levels (range: 1–4). Ten patients (45.5%) had fusion extending to L5-S1, among whom two received bilateral S2AI screws for enhanced sacropelvic fixation due to severe pelvic obliquity. Eighteen patients (81.8%) with lower extremity radicular symptoms underwent targeted foraminal decompression at a mean of 1.80 ± .6 levels per patient.

### Clinical outcomes

All clinical outcome measures demonstrated statistically significant and clinically meaningful improvement from baseline through final follow-up (Table 4).

**Table 4:** Clinical outcome scores over time.

Outcome	Pre-operative	POD 1	Final F/U	*F*-value	*P*-value	Partial η^2^
VAS back (0–10)	5.7 ± 1.0	3.5 ± 0.7^a^	1.4 ± 0.3^ab^	2,322.2	< 0.001	0.991
VAS leg (0–10)	4.8 ± 1.3	2.8 ± 0.9^a^	1.1 ± 0.4^ab^	1,486.5	< 0.001	0.986
ODI (%)	79.9 ± 9.3	51.6 ± 8.9^a^	11.7 ± 2.2^ab^	3,770.4	< 0.001	0.994
SF-36	68.6 ± 5.3	78.4 ± 5.5^a^	109.9 ± 4.9^ab^	21,736.5	< 0.001	0.999

Data presented as mean ± SD. POD 1 = post-operative day 1; F/U = follow-up. ^a^Significantly different from pre-operative (*P* < 0.001 by *post-hoc* Bonferroni). ^b^Significantly different from POD 1 (*P* < 0.001 by *post-hoc* Bonferroni).

Mean ODI improvement rate was 85.48 ± .1%. Twenty patients (90.9%) reported complete resolution of radicular symptoms by 3-month follow-up. Two patients experienced persistent but significantly improved anterior thigh numbness at final follow-up, both with pre-operative L3–4 foraminal stenosis.

### Radiographic outcomes

In addition to coronal Cobb angle, key sagittal alignment parameters (LL and SVA) were analyzed longitudinally to evaluate correction and maintenance; these results are summarized in Tables 5, 6. Mean coronal Cobb correction was 70.3%, with minimal loss of correction (1.01 ± .2°) between immediate post-operative and final follow-up. Sagittal realignment achieved physiologic lumbar lordosis restoration in all patients, with SVA normalized to < 50 mm in 20 patients (90.9%).

**Table 5:** Correction loss and maintenance rates.

Parameter	Value (mean ±SD)
Correction loss (°)	1.0 ± 1.2
Correction maintenance (%)	88.2 ± 7.3

**Table 6:** Longitudinal changes in radiographic parameters (coronal Cobb angle, lumbar lordosis, and sagittal vertical axis).

Parameter	Pre-operative	POD 1	Final F/U	*F*-value	*P*-value	Partial η^2^
Cobb angle (°)	27.81 ± 4.1	7.5 ± 3.5^a^	8.5 ± 3.8^a^	3.551	< 0.001	0.629
Lumbar lordosis (°)	24.61 ± 2.3	44.2 ± 8.6^a^	43.1 ± 8.9^a^	2.847	< 0.001	0.576
SVA (mm)	68.42 ± 8.7	22.8 ± 15.4^a^	26.3 ± 16.2^a^	2.156	< 0.001	0.507

Data presented as mean ± SD.

SVA, sagittal vertical axis.

^a^Significantly different from pre-operative (*P* < 0.001 by *post-hoc* Bonferroni).

Effect sizes are reported as partial eta-squared (partial η^2^).

### Fusion assessment

At final follow-up, solid arthrodesis (Bridwell grade I or II) was documented in all 22 patients (100%). Grade I fusion (mature trabecular bridging) was present in 18 patients (81.8%), grade II in four patients (18.2%). No pseudarthrosis, implant loosening, or rod fracture occurred. Dynamic radiographs confirmed absence of motion across instrumented segments.

### Complications

Overall complication rate was 22.7% (5/22 patients), all managed non-operatively without requiring revision surgery (Table 7). Notably, no PJK or PJF occurred despite relatively short constructs compared to traditional long-segment fusions. The two cases of adjacent segment degeneration involved mild radiographic changes at proximal unfused levels without functional impairment requiring intervention.

**Table 7:** Complication profile.

Complication (occurred)	*n* (%)	Management	Outcome
Superficial wound infection	1 (4.5)	Local wound care + oral antibiotics	Complete resolution
Deep vein thrombosis	1 (4.5)	Anticoagulation	Complete resolution
Pneumonia	1 (4.5)	Antibiotics + respiratory therapy	Complete resolution
Symptomatic adjacent segment degeneration	2 (9.1)	Physical therapy + medications	Mild residual symptoms
Total complications	5 (22.7)	All non-operative	No revisions

### Predictors of clinical outcome

Multivariate linear regression analysis identified independent predictors of ODI improvement rate at final follow-up (Table 8). In multivariable analysis, the number of degenerated PVM segments was negatively associated with ODI improvement rate (*B* = −0.11, 95% CI −0.18 to −0.04, *P* = 0.003), indicating that a greater number of degenerated PVM segments was associated with a smaller degree of ODI improvement. The number of degenerated PVM segments showed inverse correlation with outcome improvement (more extensive degeneration associated with poorer pre-operative status but similar absolute post-operative scores). Concordance between fusion extent and PVM degeneration pattern—precisely matching construct boundaries to muscle pathology—strongly predicted superior functional recovery (β = 0.386, *P* = 0.006). Traditional parameters including age, curve magnitude, and distal fixation level did not independently influence outcomes.

**Table 8:** Multivariate predictors of ODI improvement rate.

Variable	*B* (95% CI)	*P*-value	VIF
No. of degenerated PVM segments	−0.11 (−0.18 to −0.04)	0.003	1.86
Fusion-degeneration concordance	0.38 (0.12 to 0.63)	0.006	1.52
Age (years)	−0.05 (−0.15 to 0.05)	0.312	1.28
Pre-operative Cobb angle (°)	0.07 (−0.08 to 0.21)	0.521	1.43
Fusion to S1 (yes/no)	0.32 (−0.26 to 0.89)	0.278	1.67
Operative time (min)	−0.03 (−0.12 to 0.06)	0.548	2.14
Blood loss (ml)	−0.03 (−0.09 to 0.04)	0.476	1.93

*B* indicates the unstandardized regression coefficient; CI, confidence interval; VIF, variance inflation factor.

### Representative case illustration

**Figure 2:**
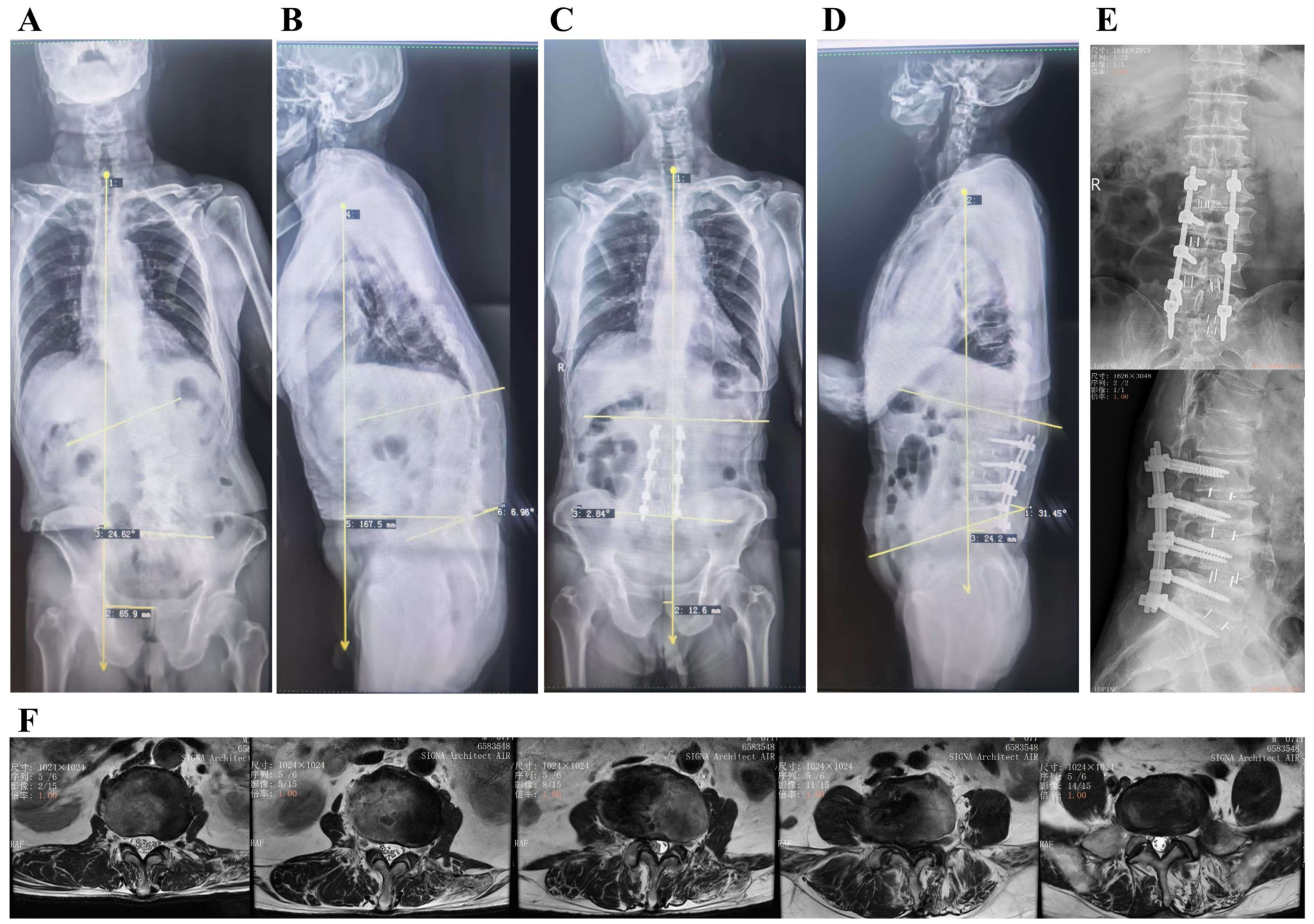
Pre-operative and post-operative radiographic and MRI findings. **(A)** Standing full-length anteroposterior radiograph of the spine demonstrating left lumbar scoliosis with a Cobb angle of 24.62° and coronal imbalance with 65.9 mm rightward deviation. **(B)** Standing full-length lateral radiograph of the spine showing loss of lumbar lordosis with kyphotic deformity, Cobb angle of 6.96°, and sagittal imbalance with 167.5 mm anterior translation. **(C)** Standing full-length anteroposterior radiograph showing excellent correction of scoliosis with well-positioned instrumentation, Cobb angle improved to 2.84°, and coronal balance restored with 12.6 mm rightward deviation. **(D)** Standing full-length lateral radiograph demonstrating restoration of sagittal alignment with Cobb angle of 31.45° and improved sagittal balance with 24.2 mm anterior translation. **(E)** Anteroposterior and lateral lumbar radiographs at 24-month follow-up showing well-positioned interbody cages and instrumentation with no screw loosening, and evidence of callus formation in the grafted regions indicating solid fusion. **(F)** Axial T2-weighted MRI of the lumbar spine demonstrating fatty infiltration of the left multifidus muscle with Goutallier grades ranging from 1 to 4 across the L1–2 through L5-S1 levels; erector spinae muscle showing Goutallier grades ranging from 2 to 4 across the L1–2 through L5-S1 levels.

A representative case demonstrating the muscle-guided selective fusion approach involved a 77-year-old male patient presenting with progressive low back pain and gait disturbance. Pre-operative standing radiographs revealed left lumbar scoliosis with a Cobb angle of 24.62° and significant coronal imbalance with 65.9 mm rightward deviation (Figure 2A). Lateral radiograph demonstrated loss of lumbar lordosis with kyphotic deformity, Cobb angle of 6.96°, and marked sagittal imbalance with 167.5 mm anterior translation (Figure 2B). Axial T2-weighted MRI assessment revealed progressive fatty infiltration of the paravertebral muscles (Figure 2F). The left multifidus muscle exhibited Goutallier grades ranging from 1 to 4 across the L1–2 through L5-S1 levels, while the erector spinae demonstrated Goutallier grades ranging from 2 to 4 across the same segmental levels. Based on our muscle-guided protocol, fusion levels were strategically selected to encompass all segments with Goutallier grade ≥3 degeneration while deliberately preserving proximal and distal zones where muscle quality remained grade ≤ 2, thereby maintaining functional dynamic stabilizers at construct boundaries. Post-operative standing radiographs obtained at final follow-up demonstrated excellent correction of scoliosis with well-positioned instrumentation, Cobb angle improved to 2.84°, and substantial restoration of coronal balance with only 12.6 mm residual rightward deviation (Figure 2C). Lateral radiograph showed restoration of sagittal alignment with Cobb angle of 31.45° and marked improvement in sagittal balance with 24.2 mm anterior translation (Figure 2D).

At 24-month follow-up, radiographs revealed well-positioned interbody cages and instrumentation with no evidence of screw loosening or implant failure, and prominent callus formation in the grafted regions indicating solid fusion (Figure 2E). The patient reported substantial clinical improvement, with ODI decreasing from 48 pre-operatively to eight at final follow-up, VAS pain score reducing from 6 to 1, and SF-36 improving from 65 to 112. No proximal junctional kyphosis or proximal junctional failure was observed during follow-up.

This case exemplifies the clinical application and favorable outcomes achievable with muscle-guided selective fusion, demonstrating substantial deformity correction (88.5% Cobb angle improvement), solid arthrodesis, excellent functional restoration, and complete absence of junctional complications through biological optimization of construct boundaries.

## Discussion

### Principal findings and clinical significance

This study presents a biology-guided paradigm for surgical planning in DLS by utilizing PVM degeneration patterns as the primary determinant of fusion level selection. The principal finding of this study is that a PVM degeneration-guided selective fusion strategy achieved a 0% incidence of PJK/PJF at a minimum 24-month follow-up despite a relatively short fusion construct. Our results demonstrate that this muscle-guided approach achieves excellent clinical outcomes—with ODI improvement of 85.4%, VAS reduction from 5.7 to 1.4, and SF-36 improvement from 68.6 to 109.9—while maintaining substantial deformity correction (mean Cobb angle correction of 70.3%) and achieving 100% fusion rate (14). Most notably, we observed zero cases of proximal junctional complications despite mean fusion extent of only 4.3 segments, challenging the traditional “longer is safer” doctrine in adult spinal deformity surgery.

### Comparison with contemporary surgical strategies

The prevailing surgical philosophy for DLS has emphasized extensive instrumented fusion to ensure robust correction and minimize mechanical failure. Smith et al.'s multicenter analysis of 147 DLS patients undergoing long-segment fusion (mean 7.2 segments) reported ODI improvement from 48 to 22 points but documented 32% incidence of PJK at 2-year follow-up (15). Our cohort demonstrated superior functional improvement (ODI improvement rate 85.4%) with complete absence of junctional complications, despite 45.5% of cases extending to S1. This divergence suggests that preserving proximal muscle quality—rather than extending fixation “one level above the deformity”—may serve as a more effective biological safeguard against junctional failure (16).

The physiologic costs of traditional approaches warrant consideration. Long-segment constructs impose substantial metabolic burden (operative time >4 h, blood loss >800 ml), prolonged hospitalization, and elevated complication rates approaching 40–50% in elderly populations. Our mean operative time (255 min) and blood loss (300 ml) represent marked reductions compared to historical controls, potentially translating to improved perioperative safety profiles in this vulnerable demographic. While their constructs were shorter, our muscle-guided approach yielded superior functional outcomes despite slightly longer constructs (4.3 segments), suggesting that precision in level selection—informed by tissue quality rather than geometric parameters alone—may optimize functional restoration (8). Similarly, Kim et al.'s Korean cohort of 52 DLS patients undergoing “strategic” 4–6 segment fusions based on sagittal vertical axis and curve flexibility reported ODI improvement from 58 to 24 with 15% PJK incidence (3). Our approach achieved substantially better functional restoration (final ODI 11.7) and eliminated junctional failures entirely, underscoring the potential additive value of incorporating muscle quality assessment into surgical decision algorithms. These comparisons highlight a critical insight: fusion extent alone does not determine outcomes (17). Rather, the biological appropriateness of construct boundaries—whether they respect or violate zones of preserved dynamic stability—appears to exert dominant influence on both symptom resolution and junctional integrity.

### Biological rationale and supporting evidence

The conceptual foundation of our approach rests on recognizing PVM degeneration not merely as a consequence of spinal pathology, but as a quantifiable marker of segmental biomechanical failure (18). The multifidus, in particular, provides primary segmental stabilization through fine motor control and proprioceptive feedback (19). Hyun et al. demonstrated that patients developing PJK exhibited significantly worse proximal PVM quality (mean Goutallier grade 2.8) compared to those maintaining junctional integrity (grade 1.6), establishing muscle quality as a independent risk factor for mechanical complications (20). Our findings extend this observation by operationalizing it prospectively: we deliberately selected fusion boundaries to preserve zones of healthy muscle (Goutallier ≤ 2) proximally and distally, avoiding iatrogenic disruption of functional stabilizers. The complete absence of PJK/PJF in our cohort, despite relatively distal constructs (45.5% to S1), provides preliminary evidence that this strategy confers protective effects.

### Biomechanical validation

Finite element modeling by Wang et al. quantified the functional consequences of fatty infiltration, demonstrating that segments with >50% adipose replacement (Goutallier ≥3) exhibit 68% reduction in axial stiffness and 73% increase in shear displacement under physiological loads (21). These biomechanical deficits translate clinically to loss of effective dynamic stabilization—the segments become “pre-failed” from a functional standpoint, unable to contribute meaningfully to load-sharing or motion control (22). Our adoption of Goutallier ≥3 as the threshold for mandatory fusion directly translates this biomechanical evidence into clinical decision-making. Segments meeting this criterion lack the mechanical capacity to maintain stability; attempting to preserve them risks persistent instability and symptom recurrence (23). Conversely, segments with preserved muscle quality (Goutallier ≤ 2) retain residual dynamic stabilization capacity that, when protected from iatrogenic surgical trauma, may buffer the mechanical transition at construct boundaries (24). This principle explains the paradoxical success of our relatively short constructs: by avoiding unnecessary extension into healthy muscle territories, we preserved functional shock absorbers that dissipate stress concentrations—the primary mechanism underlying junctional failure in traditional long constructs (25).

### Methodological strengths and clinical utility

A critical prerequisite for any biomarker-guided strategy is measurement reliability. Our excellent inter-rater agreement (κ = 0.82) validates the Goutallier classification as a reproducible tool suitable for clinical decision-making. We enhanced grading accuracy through several methodological refinements: standardizing assessment at the mid-vertebral body level where muscle cross-sectional area is maximal, evaluating both multifidus and erector spinae bilaterally with the worse side determining segment grade, and implementing formal training sessions for raters with reference image sets (26). Furthermore, our histopathologic validation—demonstrating concordance between MRI-based Goutallier grades and intraoperative biopsy specimens—confirms that grade 3 degeneration corresponds to approximately 47–63% adipocyte infiltration with associated myofiber atrophy, fibrosis, and microvascular changes (27). This structural pathology directly impairs contractile function and load-bearing capacity, providing mechanistic support for the Goutallier ≥3 threshold we employed.

Our multivariate analysis revealed that fusion-degeneration concordance—precisely matching construct boundaries to muscle pathology zones—independently predicted superior functional recovery (*B* = 0.386, *P* = 0.006), while traditional parameters including age, curve magnitude, and distal fixation level did not (28). This finding carries important implications: it suggests that biological appropriateness of level selection supersedes patient demographics or deformity severity in determining outcomes.

The inverse correlation between number of degenerated segments and ODI improvement rate (*B*=-0.412, *P* = 0.003) merits interpretation. Patients with extensive muscle degeneration presented with more severe baseline disability (stronger correlation between degenerated segments and pre-operative ODI, *r* = 0.658), yet achieved similar absolute post-operative scores as those with limited pathology (29). This pattern indicates that comprehensive addressal of degenerated territories—rather than arbitrary construct length—drives symptom resolution.

### Mechanisms of junctional protection

The complete absence of PJK/PJF in our cohort, despite nearly half extending to S1, warrants mechanistic exploration. We propose that by terminating constructs at zones of preserved muscle quality, we maintained functional stabilizers capable of dissipating sagittal bending moments and attenuating shock loads transmitted to proximal junctional bone (30), whereas traditional long constructs often extend into healthy muscle territories, necessitating iatrogenic detachment and denervation that eliminates these protective elements (31). Furthermore, when proximal junctional muscle quality is preserved, the effective stiffness gradient becomes less abrupt, as the functional construct boundaries extend beyond the instrumented levels through residual dynamic stabilization, creating a graduated mechanical transition zone rather than the sharp discontinuity that characterizes the transition from rigid instrumented segments to mobile spinal units in conventional approaches (32). Our selective approach also minimizes soft tissue disruption at unfused levels through avoidance of surgical dissection and retraction at proximal junctional levels, thereby preventing the direct muscle trauma, denervation, and devascularization that have been independently associated with PJK in prior studies. These synergistic mechanisms align with emerging evidence that junctional complications arise not from insufficient construct length *per se*, but from disruption of biological protective factors at boundaries selected without consideration of tissue quality (33).

### Study limitations and future directions

This investigation has several important limitations that merit acknowledgment. The small sample size and single-center design limit generalizability and preclude detection of rare complications, and while our zero PJK rate is striking, the confidence interval (0–15.4% by exact binomial calculation) remains wide (34). This was a retrospective single-cohort study without a control group treated with alternative fusion-level selection strategies (e.g., longer fusion constructs, alignment-target-driven planning, or conventional selective fusion based primarily on radiographic deformity) (35). The retrospective design introduces potential selection bias, though consecutive enrollment mitigates this concern, and our relatively short follow-up (mean 28.4 months) captures early mechanical failures but may miss late-onset adjacent segment degeneration or delayed junctional complications reported 5–10 years post-operatively in some series. We did not systematically compare the biology-guided selective fusion to traditional long-segment approaches in randomized fashion, and the observed benefits may reflect factors beyond muscle-based planning, including surgical technique refinements, enhanced perioperative care, or patient selection characteristics not fully captured by inclusion criteria (35). The ideal validation would involve prospective randomized controlled trials comparing muscle-guided selective fusion to matched controls receiving standard long-segment constructs, with blinded outcome assessment and sufficient power to detect differences in revision rates and junctional complications. Extension to more complex presentations—severe rigid deformities, sagittal plane dominant pathology, or frail elderly patients with multiple comorbidities—requires cautious validation, as muscle assessment may have different predictive value in these contexts (36). Bone mineral density was not systematically available for this retrospective series because DXA was not performed under a uniform protocol for all patients. Given that reduced BMD may influence fusion biology and the risk of junctional complications, the absence of standardized BMD data may have introduced unmeasured confounding. Finally, we acknowledge that muscle degeneration represents one dimension of segmental pathology, and disc height preservation, facet joint integrity, ligamentous competence, and bone quality all contribute to segmental stability, such that future iterations of biological planning algorithms should integrate multiple tissue quality biomarkers to achieve comprehensive assessment of segmental “health” rather than relying on muscle status alone (37).

### Clinical implications and future research priorities

The findings of this study suggest several important clinical implications and research directions that warrant further investigation. Our results support a paradigm evolution in surgical planning from geometry-based approaches focusing on curve magnitude and sagittal alignment toward tissue quality-based planning incorporating biological markers of segmental function (38), an evolution that parallels developments in other surgical disciplines such as oncologic margin selection guided by molecular markers rather than arbitrary distances, or fracture fixation strategies informed by bone quality assessment (39). A multi-institutional registry prospectively implementing muscle-guided selective fusion across diverse practice settings would establish external validity, capture rare complications, and enable development of refined patient selection criteria while providing long-term follow-up (minimum 5 years) to assess durability of clinical success and late mechanical failures. Head-to-head comparison with traditional long-segment fusion through pragmatic randomized trials would provide definitive evidence of superiority, equipoise, or inferiority, with trial design incorporating patient-centered outcomes, cost-effectiveness analysis, and stratification by deformity severity to identify optimal application contexts (40). Patient-specific finite element modeling comparing muscle-guided constructs to traditional long fusions would elucidate mechanical consequences of different boundary selections, while *in vivo* kinematic analysis using dynamic imaging or wearable sensors could quantify residual motion at unfused levels and validate proposed junctional protection mechanisms (41). Quantitative MRI techniques—proton density fat fraction, T2 mapping, elastography—offer potential for more precise muscle assessment (42), and machine learning algorithms trained on large imaging datasets might automate degeneration grading and predict fusion outcomes, enhancing reproducibility and clinical utility (43). Finally, if preserved muscle quality is critical to junctional protection, interventions to enhance muscle function—targeted rehabilitation, pharmacologic therapies addressing fatty infiltration, or cell-based regenerative approaches—might expand the population suitable for selective fusion or improve outcomes in patients with borderline muscle quality (44).

## Conclusion

This study introduces a biology-guided surgical strategy for degenerative lumbar scoliosis wherein fusion level selection is guided primarily by paravertebral muscle degeneration patterns rather than traditional geometric parameters. Our preliminary results demonstrate that this approach achieves excellent clinical and radiographic outcomes—comparable or superior to traditional long-segment constructs—while minimizing fusion extent, reducing operative trauma, and potentially eliminating proximal junctional complications in appropriately selected patients.

The findings challenge the prevailing “longer is safer” philosophy, suggesting that biological appropriateness of construct boundaries—whether they preserve or violate zones of dynamic stability—may exert greater influence on outcomes than fusion extent *per se*. By operationalizing muscle quality assessment into clinical decision algorithms, we translate biomechanical principles and observational risk factor data into a proactive surgical planning framework.

While these results are encouraging, they represent initial evidence from a small single-center cohort requiring validation through larger prospective studies and comparative trials. If confirmed, muscle-guided selective fusion may represent a meaningful advance in adult spinal deformity surgery, optimizing the balance between achieving necessary correction and minimizing iatrogenic harm—a fundamental principle underlying all surgical innovation.

## Data Availability

The raw data supporting the conclusions of this article will be made available by the authors, without undue reservation.

## References

[1] Asada T Simon CZ Durbas A Allen MRJ DiSilvestro KJ Hirase T . Short-segment fusion versus isolated decompression in lumbar spinal canal stenosis patients with Cobb angles over 20°. Spine J. (2025) 25:669–78. doi: 10.1016/j.spinee.2024.10.007 39505012

[2] Echt M De La Garza Ramos R Geng E Isleem U Schwarz J Girdler S . Decompression Alone in the Setting of Adult Degenerative Lumbar Scoliosis and Stenosis: A Systematic Review and Meta-Analysis. Glob Spine J. (2023) 13:861–72. doi: 10.1177/21925682221127955 PMC10240589 36127159

[3] Kim HJ Yang JH Chang D-G Suk S-I Suh SW Song K-S . Adult Spinal Deformity: Current Concepts and Decision-Making Strategies for Management. Asian Spine J. (2020) 14:886–97. doi: 10.31616/asj.2020.0568 33254357 PMC7788366

[4] Ammanuel SG Page PS Greeneway GP Ansari D Stadler JA. Early clinical outcomes and medical complications following long segment fusion for adult spinal deformity with and without three column osteotomy. World Neurosurg X. (2024) 24:100415. doi: 10.1016/j.wnsx.2024.100415 39399352 PMC11470477

[5] Shukla IY Azam F Hicks WH Hall K Akbik OS Bagley CA. Pain outcomes following long-segment thoracolumbar fusion: a three-year mixed-effects analysis. Neurosurg Rev. (2025) 48:723. doi: 10.1007/s10143-025-03822-5 41108450

[6] Lee B-J Bae SS Choi HY Park JH Hyun S-J Jo DJ . Korean spinal deformity society (KSDS). proximal junctional kyphosis or failure after adult spinal deformity surgery-review of risk factors and its prevention. Neurospine. (2023) 20:863–75. doi: 10.14245/ns.2346476.238 37798982 PMC10562224

[7] Park S-J Park J-S Kang D-H Lee C-S Kim H-J. Incidence and Risk Factors of Recurrent Proximal Junctional Failure in Adult Spinal Deformity Surgery. Glob Spine J. (2025) 15:2660–8. doi: 10.1177/21925682241308510 PMC11632719 39659051

[8] Diebo BG Shah NV Boachie-Adjei O Zhu F Rothenfluh DA Paulino CB . Adult spinal deformity. Lancet. (2019) 394:160–72. doi: 10.1016/S0140-6736(19)31125-0 31305254

[9] Xu Y Fan C Li D Qiu Y Liu Z Zhu Z. Effect of pelvic compensation capacity on proximal junctional kyphosis: a stratified analysis of pelvic tilt in adult spinal deformity surgery. J Orthop Surg. (2025) 20:675. doi: 10.1186/s13018-025-06103-5 PMC12273231 40682128

[10] Zhang C Sun R Wu X Sun X. Correlation and risk factor analysis of multifidus muscle atrophy in degenerative lumbar spondylolisthesis. Front Med. (2025) 12:1609660. doi: 10.3389/fmed.2025.1609660 PMC12187810 40568205

[11] Wang J Zhao Z Liang D Xu X Shu D. Paraspinal muscles and gluteus medius fat infiltration are both associated with lumbar disc herniation. Insights Imaging. (2025) 16:176. doi: 10.1186/s13244-025-02064-9 40810850 PMC12354410

[12] Cooley JR Jensen TS Kjaer P Jacques A Theroux J Hebert JJ. Spinal degeneration is associated with lumbar multifidus morphology in secondary care patients with low back or leg pain. Sci Rep. (2022) 12:14676. doi: 10.1038/s41598-022-18984-1 36038653 PMC9424282

[13] Patel RV Chalif JI Yearley AG Jha R Chalif EJ Zaidi HA. Impact of Adjacent Muscular Anatomic Preservation on Proximal Junctional Kyphosis and Failure. World Neurosurg. (2025) 195:123741. doi: 10.1016/j.wneu.2025.123741 39889963

[14] Yagi M Hosogane N Watanabe K Asazuma T Matsumoto M. The paravertebral muscle and psoas for the maintenance of global spinal alignment in patient with degenerative lumbar scoliosis. Spine J. (2016) 16:451–8. doi: 10.1016/j.spinee.2015.07.001 26165478

[15] Smith JS Klineberg E Lafage V Shaffrey CI Schwab F Lafage R . Prospective multicenter assessment of perioperative and minimum 2-year postoperative complication rates associated with adult spinal deformity surgery. J Neurosurg Spine. (2016) 25:1–14. doi: 10.3171/2015.11.SPINE151036 26918574

[16] Hyun S-J Kim YJ Rhim S-C. Patients with proximal junctional kyphosis after stopping at thoracolumbar junction have lower muscularity, fatty degeneration at the thoracolumbar area. Spine J. (2016) 16:1095–101. doi: 10.1016/j.spinee.2016.05.008 27217332

[17] Lafage R Schwab F Glassman S Bess S Harris B Sheer J . Age-adjusted alignment goals have the potential to reduce PJK. Spine. (2017) 42:1275–82. doi: 10.1097/BRS.0000000000002146 28263226

[18] Fortin M Lazáry À Varga PP McCall I Battié MC. Paraspinal muscle asymmetry and fat infiltration in patients with symptomatic disc herniation. Eur Spine J. (2016) 25:1452–9. doi: 10.1007/s00586-016-4503-7 26957101

[19] Bailey JF Miller SL Khieu K O'Neill CW Healey RM Coughlin DG . From the international space station to the clinic: how prolonged unloading may disrupt lumbar spine stability. Spine J. (2018) 18:7–14. doi: 10.1016/j.spinee.2017.08.261 28962911 PMC6339989

[20] Hyun S-J Bae C-W Lee S-H Rhim S-C. Fatty degeneration of the paraspinal muscle in patients with degenerative lumbar kyphosis: a new evaluation method of quantitative digital analysis using MRI and CT scan. Clin Spine Surg Spine Publ. (2016) 29:441–7. doi: 10.1097/BSD.0b013e3182aa28b0 27879506

[21] Wang W Pei B Pei Y Shi Z Kong C Wu X . Biomechanical effects of posterior pedicle fixation techniques on the adjacent segment for the treatment of thoracolumbar burst fractures: a biomechanical analysis. Comput Methods Biomech Biomed Engin. (2019) 22:1083–92. doi: 10.1080/10255842.2019.1631286 31225742

[22] Crawford RJ Filli L Elliott JM Nanz D Fischer MA Marcon M . Age- and level-dependence of fatty infiltration in lumbar paravertebral muscles of healthy volunteers. Am J Neuroradiol. (2016) 37:742–8. doi: 10.3174/ajnr.A4596 26635285 PMC7960169

[23] Peng X Li X Xu Z Wang L Cai W Yang S . Age-related fatty infiltration of lumbar paraspinal muscles: a normative reference database study in 516 Chinese females. Quant Imaging Med Surg. (2020) 10:1590–601. doi: 10.21037/qims-19-835 32742954 PMC7378097

[24] Fortin M Videman T Gibbons LE Battié MC. Paraspinal muscle morphology and composition: a 15-yr longitudinal magnetic resonance imaging study. Med Sci Sports Exerc. (2014) 46:893–901. doi: 10.1249/MSS.0000000000000179 24091994

[25] Hostin R McCarthy I O'Brien M Bess S Line B Boachie-Adjei O . Incidence, mode, and location of acute proximal junctional failures after surgical treatment of adult spinal deformity. Spine. (2013) 38:1008–15. doi: 10.1097/BRS.0b013e318271319c 22986834

[26] Shahidi B Parra CL Berry DB Hubbard JC Gombatto S Zlomislic V . Contribution of lumbar spine pathology and age to paraspinal muscle size and fatty Infiltration. Spine. (2017) 42:616–23. doi: 10.1097/BRS.0000000000001848 27517512 PMC5303569

[27] Sun D Liu P Cheng J Ma Z Liu J Qin T. Correlation between intervertebral disc degeneration, paraspinal muscle atrophy, and lumbar facet joints degeneration in patients with lumbar disc herniation. BMC Musculoskelet Disord. (2017) 18:167. doi: 10.1186/s12891-017-1522-4 28427393 PMC5399427

[28] Yagi M King AB Boachie-Adjei O. Incidence, risk factors, and natural course of proximal junctional kyphosis: surgical outcomes review of adult idiopathic scoliosis minimum 5 years of follow-up. Spine. (2012) 37:1479–89. doi: 10.1097/BRS.0b013e31824e4888 22357097

[29] Kim HJ Iyer S Zebala LP Kelly MP Sciubba D Protopsaltis TS . Perioperative neurologic complications in adult spinal deformity surgery: incidence and risk factors in 564 patients. Spine. (2017) 42:420–7. doi: 10.1097/BRS.0000000000001774 27398890

[30] Hart RA McCarthy I Ames CP Shaffrey CI Hamilton DK Hostin R. Proximal junctional kyphosis and proximal junctional failure. Neurosurg Clin N Am. (2013) 24:213–8. doi: 10.1016/j.nec.2013.01.001 23561560

[31] Lau D Clark AJ Scheer JK Daubs MD Coe JD Paonessa KJ . Proximal junctional kyphosis and failure after spinal deformity surgery: a systematic review of the literature as a background to classification development. Spine. (2014) 39:2093–102. doi: 10.1097/BRS.0000000000000627 25271516

[32] Bridwell KH Lenke LG Cho SK Pahys JM Zebala LP Dorward IG . Proximal junctional kyphosis in primary adult deformity surgery: evaluation of 20 degrees as a critical angle. Neurosurgery. (2013) 72:899–906. doi: 10.1227/NEU.0b013e31828bacd8 23407291

[33] Kim HJ Bridwell KH Lenke LG Park MS Ahmad A Song K-S . A proximal junctional kyphosis results in inferior SRS pain subscores in adult deformity patients. Spine. (2013) 38:896–901. doi: 10.1097/BRS.0b013e3182815b42 23232215

[34] Yagi M Akilah KB Boachie-Adjei O. Incidence, risk factors and classification of proximal junctional kyphosis: surgical outcomes review of adult idiopathic scoliosis. Spine. (2011) 36:E60–8. doi: 10.1097/BRS.0b013e3181eeaee2 21192216

[35] Ames CP Smith JS Scheer JK Bess S Bederman SS Deviren V . Impact of spinopelvic alignment on decision making in deformity surgery in adults: a review. J Neurosurg Spine. (2012) 16:547–64. doi: 10.3171/2012.2.SPINE11320 22443546

[36] Schwab F Ungar B Blondel B Buchowski J Coe J Deinlein D . Scoliosis research society—schwab adult spinal deformity classification: a validation study. Spine. (2012) 37:1077–82. doi: 10.1097/BRS.0b013e31823e15e2 22045006

[37] Klineberg EO Passias PG Jalai CM Worley N Sciubba DM Burton DC . Predicting extended length of hospital stay in an adult spinal deformity surgical population. Spine. (2016) 41:E798–805. doi: 10.1097/BRS.0000000000001391 26679876

[38] Protopsaltis T Schwab F Bronsard N Smith JS Klineberg E Mundis G . The T1 pelvic angle, a novel radiographic measure of global sagittal deformity, accounts for both spinal inclination and pelvic tilt and correlates with health-related quality of life. J Bone Jt Surg. (2014) 96:1631–40. doi: 10.2106/JBJS.M.01459 25274788

[39] Glassman SD Berven S Bridwell K Horton W Dimar JR. Correlation of radiographic parameters and clinical symptoms in adult scoliosis. Spine. (2005) 30:682–8. doi: 10.1097/01.brs.0000155425.04536.f7 15770185

[40] Bess S Line B Fu K-M McCarthy I Lafage V Schwab F . The health impact of symptomatic adult spinal deformity: comparison of deformity types to United States population norms and chronic diseases. Spine. (2016) 41:224–33. doi: 10.1097/BRS.0000000000001202 26571174 PMC4718181

[41] Hallager DW Hansen LV Dragsted CR Peytz N Gehrchen M Dahl B . A comprehensive analysis of the SRS-schwab adult spinal deformity classification and confounding variables: a prospective, non-US cross-sectional study in 292 patients. Spine. (2016) 41:E589–97. doi: 10.1097/BRS.0000000000001355 26656058

[42] Ranson CA Burnett AF Kerslake R Batt ME O'Sullivan PB. An investigation into the use of MR imaging to determine the functional cross sectional area of lumbar paraspinal muscles. Eur Spine J. (2006) 15:764–73. doi: 10.1007/s00586-005-0909-3 15895259 PMC3489434

[43] Galbusera F Wilke H-J Brayda-Bruno M Costa F Fornari M. Influence of sagittal balance on spinal lumbar loads: a numerical approach. Clin Biomech. (2013) 28:370–7. doi: 10.1016/j.clinbiomech.2013.02.006 23489477

[44] Kjaer P Bendix T Sorensen JS Korsholm L Leboeuf-Yde C. Are MRI-defined fat infiltrations in the multifidus muscles associated with low back pain? BMC Med. (2007) 5:2. doi: 10.1186/1741-7015-5-2 17254322 PMC1796893

